# Brain Computer Interfaces and Communication Disabilities: Ethical, Legal, and Social Aspects of Decoding Speech From the Brain

**DOI:** 10.3389/fnhum.2022.841035

**Published:** 2022-04-21

**Authors:** Jennifer A. Chandler, Kiah I. Van der Loos, Susan Boehnke, Jonas S. Beaudry, Daniel Z. Buchman, Judy Illes

**Affiliations:** ^1^Bertram Loeb Research Chair, Faculty of Law, University of Ottawa, Ottawa, ON, Canada; ^2^Faculty of Law, University of Ottawa, Ottawa, ON, Canada; ^3^Centre for Neuroscience Studies, Queen’s University, Kingston, ON, Canada; ^4^Institute for Health and Social Policy (IHSP) and Faculty of Law, McGill University, Montreal, QC, Canada; ^5^Centre for Addiction and Mental Health, Dalla Lana School of Public Health, Krembil Research Institute, University of Toronto Joint Centre for Bioethics, Toronto, ON, Canada; ^6^Division of Neurology, Department of Medicine, University of British Columbia, Vancouver, BC, Canada

**Keywords:** brain computer interface, BCI, communication, law, neuroethics, augmentative and alternative communication, AAC

## Abstract

A brain-computer interface technology that can decode the neural signals associated with attempted but unarticulated speech could offer a future efficient means of communication for people with severe motor impairments. Recent demonstrations have validated this approach. Here we assume that it will be possible in future to decode imagined (i.e., attempted but unarticulated) speech in people with severe motor impairments, and we consider the characteristics that could maximize the social utility of a BCI for communication. As a social interaction, communication involves the needs and goals of both speaker and listener, particularly in contexts that have significant potential consequences. We explore three high-consequence legal situations in which neurally-decoded speech could have implications: *Testimony*, where decoded speech is used as evidence; *Consent and Capacity*, where it may be used as a means of agency and participation such as consent to medical treatment; and *Harm*, where such communications may be networked or may cause harm to others. We then illustrate how design choices might impact the social and legal acceptability of these technologies.

## Introduction

Communication is central to social and political inclusion, to interacting with and influencing one’s environment, to transmitting thoughts and decisions to others, and, at a fundamental level, to accessing and to contributing to the store of knowledge and ideas that make up human cultures. For example, the classic formulation of legal decision-making capacity includes the ability to communicate a choice (Appelbaum and Grisso, 1995), underscoring the importance of communication to self-determination. The inability to communicate can also put people at risk of maltreatment because of their reduced ability to report it (e.g., Brownlie et al., 2007). The importance of communication technologies is recognized by the 2008 UN Convention on the Rights of Persons with Disabilities (CRPD) (United Nations General Assembly, 2006), which obligates states to accept and facilitate the use of augmentative and alternative communication (AAC) technologies and other accessible means and formats of communication on the grounds of equality, social inclusion, freedom of expression and access to justice. The aim of such technologies is not to normalize people with communication disabilities by imposing non-disabled modes of communication on them (Silvers, 1998). Instead, AAC technologies increase their levels of functioning by rendering communication channels and environments more universally accessible.

Communication impairments are highly varied (Box 1), and many AAC tools and techniques have been devised to respond to the needs and goals of people with these impairments (Box 2). One of the most well-known examples of their use was by the late physicist Stephen Hawking, who lived for many years with progressive motor neuron disease. Hawking used a series of computer programs through which he could operate switches to select phrases from predictive word generating software first using his hand, then a sensor on his cheek (Medeiros, 2015). As this became less efficient near the end of his life, he collaborated with Intel to develop ways to use eye tracking or electroencephalography (EEG) signals to select phrases, though Hawking was never able to adopt them (Medeiros, 2015). People with locked-in syndrome have also actively contributed to AAC research, and a 2015 French survey describes how this population uses eye movements, and sometimes other bodily movements where these abilities are preserved to operate AAC devices (Lugo et al., 2015). The efficiency of speech production remains limited, and eventually eye gaze control can fail in certain syndromes. Ultimately, a system that could decode imagined but not vocalized speech directly from the brain would be a beneficial alternative (Bocquelet et al., 2016; Cooney et al., 2018; Pandarinath and Ali, 2019).

**Box 1. Types of communication impairment.**

Bilateral communication requires both the receptive and expressive abilities, each of which includes a range of sensory, cognitive, linguistic, and motor functions. The mixture of specific functional impairments varies and affects the suitability of each type of AAC to a given user’s goals and needs. Examples of each are provided below (Cummings(ed.), 2013).

Developmental: Cerebral palsy, severe autism spectrum disorder, intellectual disability, severe dyspraxia and developmental dysarthria

Acquired: Traumatic brain injury, high-level spinal cord injury, stroke leading to aphasias and dysarthria

Degenerative: Amyotrophic lateral sclerosis (ALS), multiple sclerosis, dementia.

**Box 2. Augmentative and alternative communication (AAC).**

Augmentative and alternative communication (AAC) (Loncke, 2011; Cook et al., 2019) uses a set of tools and methods to address communication problems that may be developmental or acquired at any point during life. *Unaided* or *body-based* communication includes gestures, facial expressions, eye-gaze, manual signing, or finger-spelling. *Aided* communication ranges in technological complexity from low-tech communication boards containing letters, symbols or pictures, to more complex electronic systems including speech-generating devices (SGD) to produce pre-recorded or synthesized speech output. Word completion/prediction systems can increase the speed of written output. Users may need to access AAC devices in different ways, depending upon their motor abilities. Items may be selected by pointing with fingers, using a laser pointer mounted to the head, eye gaze tracking or using cursors projected on glasses that respond to head movements. Scanning systems allow a user to blink, vocalize or make a gesture such as a cheek movement to select an item among those being presented in a sequence. More recently, brain-computer interfaces offer techniques to access AAC systems by detecting neural activity using electroencephalography (EEG), event-related potentials (ERPs), and more recently neuroimaging such as functional magnetic resonance imaging (fMRI) and functional near-infrared spectroscopy (fNIRS). Another developing strategy is based on a neuroprosthesis that can decode imagined speech directly from neural signals in brain areas involved in speech generation, raising the hope for a system that allows more naturalistic interaction on the more rapid timescale of human conversation. This type of neuroprosthesis may not be well-suited to those who have never spoken from birth or whose speech-production impairments extend beyond motor impairment (e.g., severe intellectual disability).

Progress has been made toward this end. Speech that is heard can be decoded and resynthesized in an intelligible way from activity in the auditory areas of a subject’s brain (Akbari et al., 2019). Audible or mimed speech has also been decoded from neural activity (e.g., Anumanchipalli et al., 2019; Herff et al., 2019; Angrick et al., 2020; Dash et al., 2020), at speeds consistent with normal communication (e.g., Moses et al., 2019; Makin et al., 2020). Progress has also been made on decoding imagined speech (i.e., produced by imagining speaking but without vocalizing or activating speech articulators) from neural activity (see e.g., Martin et al., 2019; Angrick et al., 2020; Cooney et al., 2020; Dash et al., 2020; Tamm et al., 2020). The private sector is also pursuing these possibilities (Robertson, 2019), with the goal of controlling devices using brain activity, also a goal of Elon Musk’s Neuralink (Lopatto, 2019). This could possibly offer an additional communication option for those with severely limited voluntary motor function. It also might serve to allow for more rapid and naturalistic communication even for those who have sufficient preserved motor function to use existing forms of AAC. Indeed, Moses et al. (2021) recently demonstrated that full words and sentences could be decoded using deep learning algorithms from electrical activity in the sensorimotor cortex of an individual with paralysis and anarthria who was attempting, unsuccessfully, to produce intelligible speech. When combined with natural language modeling to predict probable next words, decoding was achieved at 15 words per minute with a 25% error rate. This raises possibilities for new types of communication neuroprostheses and, along with them, ethical, legal and social considerations for their use in high consequence communications.

Many features are important in a communication technology such as its efficiency, flexibility, ease of use, cost, and compatibility with the communicative contexts in which it will be used. Some communication contexts are of very high potential consequence to the speaker and to others, and neurotechnology-mediated communication poses both opportunities and vulnerabilities to the users of the technology, and importantly to their communication partners.

To explore these issues, we assume that it will one day be possible to detect imagined speech in some people with severe motor impairments. Ethico-legal analyses that anticipate future developments can be valuable in identifying relevant considerations during the developmental phase of technologies, but they also risk missing the mark if the presumptions about the capabilities and future evolution of the technologies are incorrect (Racine et al., 2014). With this limitation in mind, we focus on the use of neurotechnology to decode imagined speech and consider two areas of central importance to social and legal practices important to citizenship, personhood and justice: (i) legal testimony and (ii) capacity and consent. We then turn to (iii) risks of harm potentially associated with the use of such communication technologies. We conclude with comments on the importance of considering these kinds of high consequence communication contexts from the outset in designing communication neurotechnologies. For the technologies to offer the maximal possible benefit in terms of supporting users’ self-determination and inclusion, they should be able to satisfy the requirements of the most significant communication contexts and not just day-to-day communications.

## Three Dimensions of Communication Through Detection of Imagined Speech

### Testimony: Communication as Evidence

The ability to communicate is central to being able to contribute to the shared knowledge base of a person’s community, which in turn enriches the diversity of information and voices available. Communication is also central to whether a person has access to justice as a witness or litigant in legal proceedings. In this section, we focus on issues related to the ability to testify in court, and how neurotechnology to detect imagined speech might challenge existing practices.

People with disabilities are at greater risk of abuse than those without disabilities, with risk varying by disability, age, living environment, and gender (Perreault, 2009; Hughes et al., 2011; Khalifeh et al., 2013; Basile et al., 2016). Few researchers have studied the criminal victimization of people with communication disabilities in particular. However, Bryen, Carey and Frantz surveyed adults who use AAC, and found that nearly half (45% of 40 respondents) reported having experienced crime or abuse (Bryen et al., 2003). As a caveat, most had co-occurring physical disabilities that, by increasing the risk of victimization, could explain the high rate reported. The most common forms of abuse were theft, physical threats and attacks, and unwanted sexual touching. Perpetrators were usually known, with professional staff (personal assistants, teachers, medical personnel) most often involved, followed by friends/acquaintances and then family members. In these cases, people with severe communication disabilities are vulnerable in part because they have difficulty reporting the abuse (White et al., 2015).

Communication disabilities pose challenges at various stages of the legal process, from initial difficulty in reporting a crime to exclusion from testifying if the legal system regards a person as lacking testimonial capacity due, for example, to co-occurring mental disability. Together this makes people easy prey for abuse (*R. v. D.A.I*, 2012). The Supreme Court of Canada has noted that preventing people with mental disabilities from testifying in court could “effectively immunize an entire category of offenders from criminal responsibility for their acts and […] further marginalize the already vulnerable victims of sexual predators” (*R. v. D.A.I*, 2012, para 67). The exclusion from testifying also represents a limitation on the enjoyment of important dimensions of citizenship, such as being heard in legal proceedings and benefitting from the equal protection of the law.

The importance of accommodating witnesses’ disabilities is about more than just access to a witness stand. Some argue that by privileging certain modes of testimonial communication to the exclusion of others, evidence law declares who is a capable or incapable witness, and contributes to the social construction of people with disabilities as inferior more generally (MacCrimmon, 1991; Taslitz, 1998-1999; Ziv, 2007) and feeds stereotypes about their unreliability as witnesses (Benedet and Grant, 2012, 2013; Barnes, 2016; *R. v. Slatter*, 2019, 2020).

While broad legal attention to disability related communication obstacles is relatively recent (e.g., *R. v. D.A.I*, 2012; United Nations General Assembly, 2006; *R. v. Slatter*, 2019, 2020) justice systems have long had to contend with communication problems. The right to an interpreter for someone who does not understand or speak the language of the proceedings is enshrined in national and international human rights instruments (United Nations General Assembly, 1996; *Canadian Charter of Rights and Freedoms*, 1982). Beyond problems of language differences, many legal systems can, in principle, accommodate communication disabilities and alternative methods of communication. For example, the *Canada Evidence Act* states that “[i]f a witness has difficulty communicating by reason of a physical disability, the court may order that the witness be permitted to give evidence *by any means* that enables the evidence to be intelligible” [emphasis added] (*Canada Evidence Act*, 1985). However, legal systems have yet to fully grapple with how to make the witness stand equally accessible to people with disabilities. In Canada, for instance, virtually no case law exists applying the above-mentioned provision of the *Evidence Act* to AAC or in ways that would substantially challenge traditional modes of collecting testimonies. There has been more flexibility in relation to accommodations that do not fundamentally alter the abilities expected of witnesses, such as changing the physical settings for giving testimony (e.g., *Criminal Code of Canada*, 1985, sections 486.1-3 and 715.2). In addition, the justice system has been criticized for showing a lack of imagination on how to accommodate communication obstacles faced by people with intellectual disabilities (Benedet and Grant, 2012, 2013; Beaudry, 2016). Judicial encounters with AAC technologies outside Canada have not always gone smoothly.

In the case of *Byndom v. State* (2001), the accused was prosecuted for the sexual assault of the complainant–a woman with cerebral palsy and intellectual disabilities. She communicated using a Dynavox computer, which allowed her to touch a computer screen or type words on a keyboard to generate synthesized speech. It also allowed particular words and phrases to be programmed into the computer’s memory and represented by icons on the screen and grouped on separate pages for ease of use in different situations. A rehabilitation services provider testified that she had used a series of yes/no questions to learn what information the complainant wished to have programmed for use in court. The defense vigorously resisted the complainant’s use of the Dynavox device to testify in court on the basis that the pre-programmed statements were inadmissible hearsay (i.e., a report by a witness of what another person not present has previously said). The court accepted this argument and ruled that the preprogrammed statements were inadmissible. Answers given to yes/no questions were, however, ruled acceptable. The defendant was convicted but appealed on the basis that his ability to cross-examine the complainant was hampered. On appeal the court upheld the conviction, stating that the use of the Dynavox did not constitute hearsay where the user was present and could directly answer questions with the words and phrases she wished.

Courts generally now recognize a duty to provide reasonable accommodation of disabilities, and this will not necessarily violate a defendant’s right to confront an accuser even if cross-examination of the accuser is affected to some extent (*In re McDonough*, 2010). However, it may sometimes not be possible to accommodate a communication disability. In *R. v. W.H.*, the accused was found unfit to stand trial after aphasia caused by a stroke affected his language comprehension.

“A trial is pre-eminently an exercise in communication. It is linguistic. Decisions may turn on a phrase, a nuance of language, an inflection. These are the bases for findings of credibility, intention, memory, or other issues relevant to the case. This process of communication in the course of a trial requires sophisticated cognitive and social processing. When a witness or a participant in the trial does not understand the language of the trial, an interpreter who does understand, acts as the bridge between the person who does not speak the language and those who do. In Mr. W.H.’s case, there is no interpreter. No one speaks the language his brain now speaks” (*R. v. W.H*, 2006).

The question of whether a given form of communication assistance is a reasonable and therefore legally obligatory accommodation is an important question. There is a risk that in the well-intentioned enthusiasm for a solution, an unreliable method will be accepted. Facilitated Communication (FC) offers a cautionary example. FC is a controversial technique in which a human facilitator physically supports the arm or hand of a person with autism or another communication disability to help them point to letters, pictures or objects on a keyboard or communication board (Hemsley et al., 2018). Due to concerns that the messages are influenced by the facilitator, organizations such as the American Speech-Language-Hearing Association have taken the position that the technique should not be used (American Speech-Language-Hearing Association [ASHA], 2018). FC has been implicated in serious harmful consequences, including unreliable accusations of sexual and other abuse (Lilienfeld et al., 2014; *Minister of Health and Wellness v. Murphy*, 2016).

Forms of communication assistance vary in their degree of transparency. In theory, language interpretation is highly transparent—it proceeds according to broadly shared and publicly known linguistic rules. Another person who knows the two languages (spoken or sign) could easily verify the fidelity of the translation. Other devices use methods of interpretation that are observable and easily understood, such as observing the user of an AAC device touching icons to generate synthesized speech or using eye gaze to select letters. Newer technologies relying upon the detection of neural activity to operate an AAC, or even more ambitious, to detect imagined speech, are not transparent to the observer. In addition, the inclusion of AI-based predictive algorithms to try to enhance efficiency will add another layer of uncertainty about whether the technology is faithfully transmitting the user’s own expressions or is instead delivering something closer to facilitated communication. Since technologies that can detect imagined speech have the greatest potential to facilitate communication in real-time for people with severe motor impairments, advance consideration of how to demonstrate reliability for high-stakes communication would be wise. The inclusion of a means for the user to endorse or reject the decoded output through a distinct and specific neural task after each utterance might provide some assistance.

### Consent and Capacity: Communication as Means of Agency and Participation

Personhood is complex concept with a lengthy philosophical and social history, but often refers to two sets of related criteria: a set of abilities or features that must be possessed to be a person, and the social recognition that someone is a person (Sample et al., 2019). Communication is a critical feature in the experience of personhood, as it is so important for agency and for relations with others. As Vidal points out, “[t]he agony of incommunicability and the pain of not being able to touch their loved ones is a recurrent theme in [locked in state] testimonies” (Vidal, 2020). In this section, we focus on self-determination and choice, where the ability to communicate allows a person to make and express fundamental personal decisions that others are legally obligated to respect and allows one to obtain assistance from others to meet needs and wishes. Communication as a means of agency is crucial because the capacities for individual autonomy, agency and participation are culturally highly valued, to the point of often making them conditions of full membership in our legal and political communities (Beaudry, 2021).

The ability to communicate is often conflated, however, with the ability to be an autonomous agent. For instance, the standard formulation of medical decision-making capacity includes the abilities to understand relevant information, to appreciate the situation and its likely consequences, to manipulate information rationally, and to *communicate* a choice (Appelbaum and Grisso, 1995). Communication is often taken to be an implicit requirement for both assessing a person’s capacity as well for them to express a choice (Appelbaum and Grisso, 1995; White et al., 2015; Peterson, 2019). Cognitive function is usually preserved in locked-in syndrome (LIS)—a condition in which a person has lost all motor function except perhaps control over eye gaze and blinking. However, Bernat suggests that “primitive communication systems that restrict LIS patients’ responses to ‘yes’ or ‘no’ answers, such by decoding vertical eye or eyelid movements, are inadequate to allow patients to achieve full decision-making” for critical decisions such as discontinuation of life-sustaining therapy, which requires detailed, repeated and effective communication (Bernat, 2020). The law demands a higher standard of capacity for decisions that are more complex and consequential, and Bernat suggests that this should also include a higher standard of effective and nuanced communication (Bernat, 2020).

To be sure, the ability to communicate is central to autonomy and self-determination—the ability to exercise free choice with respect to one’s acts and states—and to political, economic and social participation. However, it is important to disentangle scientific and legal difficulty in *reaching* an individual’s expressions of agency from that agency itself. The capacity for autonomy includes the psychological abilities to formulate a concept of the good life and to exercise the self-control to pursue it to some degree. The idea that communication is a *sine qua non* feature of the capacity for autonomy is more problematic. People with disabilities may well experience difficulties in communicating their choices due to environmental obstacles or impairments, but this is not the same thing as the inability to choose. Rather the obstacles or impairments make their autonomous choices inaccessible to others. A better starting point is one where everyone is assumed to have some level of autonomy, and the focus is on how best to support the expression of that autonomy given differences in abilities (e.g., Silvers and Francis, 2010; Wong, 2010). This is not to suggest that every human being has the capacity for autonomy, but only to underscore the need to reject any starting assumption that people with communication, intellectual or other kinds of disability do not have that capacity.

There has been a growing legal acceptance that societies should respond to communication disabilities with supports and accommodations, that they should pursue continued improvements in ACC developments, and that policymakers should experiment with new communication modes. Technological changes and legislative changes to promote the inclusion and empowerment of people with disabilities such as the *Americans with Disabilities Act* of 1990 together played a crucial role in propelling forward the field of AAC (Wendt and Lloyd, 2011). At the international level, the UN CRPD (United Nations General Assembly, 2006) commits countries to ensure that people with disabilities can exercise the right to freedom of expression, including “the freedom to seek, receive and impart information and ideas” including by “accepting and facilitating the use of…augmentative and alternative communication…in official interactions.” (Article 21). The CRPD also declares that people with disabilities have equal legal capacity and requires signatories of the Convention to provide access by persons with disabilities to the support they may require in exercising their legal capacity (Article 12).

This obligation to provide support to exercise legal capacity entails government responsibility to encourage the development of and access to technologies that might establish richer channels of communication so that people can establish their capacity to make personal decisions of both private and public significance. Technologies that allow a more effective communication channel might expand the scope of self-determination for patients with severe communication impairments both by allowing them to satisfy legal capacity tests and, practically, to express their personal choices. They would also foster inclusive political deliberations that are more likely to produce fairer outcomes (Young, 1991). Inclusive communication channels are needed to realize the right to democratic participation through voting. Legal struggles over the use of AAC by people claiming their right to vote illustrate the challenges faced by people with communication disabilities (Domínguez Rubio and Lezaun, 2012; Vidal, 2020).

A key legal question for the future is whether consent via communication neurotechnology that detects imagined speech will be treated as legally effective. This will be important to the speaker who wishes to make fundamental personal decisions about matters as varied as medical care, testamentary dispositions, contracting, voting, and sexual intimacy. It is also important to others whose actions taken on the basis of that consent would otherwise constitute torts or crimes. In Canada, some of the contexts in which the individual must give valid first-person consent are medically assisted dying (*Criminal Code of Canada*, 1985, section 241.2), living donation of an organ like a kidney (*Trillium Gift of Life Network Act*, 1990) and sexual contact. If a physician or other person interferes with the body of a disabled person in one of these ways based on consent via communication neurotechnology, this would constitute a crime if the consent were not accepted as valid. Often the law offers a defense where there was a reasonable but mistaken belief in consent, but this typically requires that reasonable steps be taken to verify consent, and it would need to be clarified if and how one could do this using communication neurotechnology.

Even if the potential of communication neurotechnology is promising, a legal culture anchored in traditional modes of communication tailored by and for non-disabled people may be reluctant to accept complex technologies where the reliability of the interpretation is hard to verify. Awareness of risks and actions to mitigate them are important, as we discuss in the next section. However, the conflation of communication capacity with the capacity for autonomy runs the risk of too hasty a rejection, or too suspicious an attitude vis-à-vis these emerging technologies in the name of risk management.

### Communication as Harm

In this section, we examine the legal dimensions of potential harms associated with communication technologies. While promising in terms of enhancing testimonial abilities, agency and social participation, communication neurotechnology also carries a risk of harm to its users or of rendering its users responsible for harming others. This includes privacy issues and responsibility for communication-related civil and criminal legal wrongs, such as defamation, threats and harassment, communication of hate speech, child pornography, official secrets or incitement to terrorism. If a neurotechnology is an intermediary in such a communication, when should the user be responsible for it, and what protections should be offered to users against harms to their own interests?

From a legal perspective, responsibility usually attaches to voluntary capable acts. As Rainey and colleagues discuss, it is not clear whether communication technologies that detect imagined speech could distinguish between inner speech that one intends to vocalize and that which one does not (Rainey et al., 2019). On this point, current technology relies upon neural signals associated with the effort to move the vocal apparatus (Chang and Anumanchipalli, 2019; Robertson, 2019; Angrick et al., 2020). Thus the possibility of picking up inner speech that is not associated with articulatory effort appears to be low at present. A second problem is the accuracy of the content decoded from the user who is attempting to communicate. However, one can still be liable in negligence for harmful involuntary or incapable acts if the harm was reasonably foreseeable at an earlier point when the person was capable of choosing to take steps to avoid it. As a result, a person may be held negligent for using a communications neurotechnology that is unreliable under conditions where that use poses a reasonably foreseeable risk of harm. Note that a person need only behave reasonably, and the use of an imperfect prosthesis by someone without communication alternatives is likely to be viewed as reasonable much of the time.

Design choices could help to address problems with voluntariness and accuracy. Helpful design features could include (1) controls that activate and deactivate the detection of imagined speech so that inner speech is transmitted only when desired, (2) feedback mechanisms that allow a person to hear and approve communications before they are transmitted, and (3) signals that a person can use to rapidly indicate error after a communication has been transmitted (Rainey et al., 2019). One way in which the control of the device could be set up so that it is functional only when desired is through the use of brain-switches (Han et al., 2020). A brain-switch involves the use of two distinct neural signals, one to indicate the intention to use a brain-computer interface (BCI) (activating or de-activating the BCI control state) and a second set of neural signals used to interpret the user’s communication objectives (Rainey et al., 2019). More is required though. As Maslen and Rainey observe (Maslen and Rainey, 2021), the content of natural speech is not often fully consciously planned but instead emerges concretely in the process of speaking. Furthermore, the use of predictive methods to try to speed up decoding would introduce another uncontrolled element. This would suggest that the kind of control offered by a brain-switch that activates the decoding mode would be insufficient. Instead, something more like a mechanism to hear and approve a message prior to transmission or the capacity to use an error signal after the fact would be needed.

Although unlikely to be used for serious criminal purposes, communication neurotechnologies might still play a part in injurious communication raising some novel and as yet unresolved questions about responsibility. An example would be a BCI used to control a networked computer. People accused of child pornography offenses sometimes claim that the illegal material was accidentally downloaded (US v. Froman, 2004; *R. v. Missions*, 2005; US v Gourde, 2006). In these cases, the credibility of the claim that the download was accidental is assessed on the basis of factors such as the number and complexity of the steps required to access the material, the volume of illegal material downloaded, actions such as renaming and filing the material, and so on. BCI-enabled interactions with a computer to download illegal material could be assessed according to similar criteria. With respect to illegal communications such as threats or child luring, for example, one can expand on this reasoning to suggest the use of a BCI to select multiple phrases, words or letters in a communication is more likely to be viewed as deliberate and voluntary than one letter or word.

Child pornography offenses also highlight another legal responsibility-related dimension of BCI when used to control a computer. Here again, people accused of child pornography offenses also sometimes claim that malware or malicious hacking is the explanation for the presence of illegal material (e.g., *R. v. Jones*, 2019). This highlights the issue of cybersecurity in the use of communication neurotechnologies, particularly when networked. The possibility of malicious outside exploitation of communication neurotechnologies complicates responsibility attribution, but also represents a distinct source of harm to users whose control over devices may be undermined and whose personal data may be accessed and used by others (Landau et al., 2020). It is likely that an eventual communication neurotechnology that detects imagined speech would be used to interact with networked computers as the detection of speech would allow for rapid composition of messages and search engine input among other things.

Prosthetic devices that record data may expose users to a potential means that can be used to impeach their credibility in later legal disputes, raising a potential harm to the user to which other speakers are not necessarily exposed. This tension is exemplified in *State of Ohio* v *Ross Compton* in which a man was found guilty of arson and insurance fraud after a fire at his home. He claimed that he was asleep when his house caught fire, and that he packed some belongings in a suitcase and bags when he awoke, broke the window and threw the bags out before climbing out himself and taking the bags to his car. Police sought and obtained a search warrant for the electronic data stored on his pacemaker, which showed heart rate, pacemaker demand, and cardiac rhythm before, during and after the fire. A cardiologist found it “highly improbable” Compton would have been able to do all of the things he claimed. Compton sought to have his pacemaker data excluded as violation of his constitutional rights, and as protected by doctor-patient privilege. These claims were both rejected in March 2019, however Compton died pending a ruling in his appeal of this decision (Pack, 2020).

It is not presently clear what sort of record would be preserved by a communication neurotechnology and for how long. However, the implications of recording and preserving user data should be considered when designing these technologies so that users do not become unwittingly exposed to risks not faced by others or policed in ways other citizens are not.

## Way Forward

In this last section, we apply a disability perspective to the ongoing development of communication neurotechnology. The social model of disability, which has informed mainstream legal conceptualizations of disability, understands disability as a social phenomenon, rather than a physiological dysfunction. According to this model, disabilities are not to be equated to impairments, but instead refer to the social obstacles faced by people who happen to be cognitively or physically different from the average non-disabled person. Influential mixed models of disability such as the World Health Organization’s biopsychosocial model (World Health Organization, 2001) include a physiological dimension to disability, but there is now a wide consensus that disabilities cannot be understood apart from the social context. This social lens suggests two important points for the future development of, and standards, for communication neurotechnologies.

First, communication neurotechnologies must be developed in an inclusive manner. This is reflected in general user-centered design (UCD) principles, which rely upon the involvement of users in design and development as well as upon a focus on the key usability concepts of effectiveness, efficiency and user satisfaction. While there have been some recent steps toward specifying principles and approaches for BCI (Choi et al., 2017; Garro and McKinney, 2020; IEEE, 2020; Kubler et al., 2020), leading researchers in BCI usability recently argued that attention to usability and accessibility of BCIs continues to be inadequate and this is an impediment to translating BCI research from the laboratory to the field (Kubler et al., 2020). Kubler and colleagues suggest that one of the reasons for the failure to include end-users throughout BCI research and development is the greater complexity of involving people with severe speech and physical impairments as opposed to volunteers without impairments (Kubler et al., 2020). Attention is now being paid to this and work on how to adapt AAC techniques to obtain feedback from people with severe speech and physical impairments will be important (Peters et al., 2016). Fortunately, research into BCI user opinions, priorities and experiences is growing, some of which includes people with severe impairments (Blain-Moraes et al., 2012; Kögel et al., 2019).

Second, policymakers and scientists must approach communication as a social interaction rather than merely an individual action (Chandler et al., 2021). The needs and goals of the primary user are inextricably linked to the needs and goals of their interlocutors, who need to be able to judge both the voluntariness and the accuracy of a decoded communication This is important because errors may pose real harms to the primary user and to others. In addition, this relational aspect of the technologies is also illustrated by the need to consider how they might allow others to access and manipulate the device and its stored data. Therefore, the roles and needs of others, interacting with primary users, must be central to design from the outset. In particular, communication neurotechnology should be evaluated for a range of communicative contexts, and design choices made to maximize their suitability. Some of the design elements mentioned above, such as brain switches for on/off control, playback mechanisms, endorsement/veto signals, transparency about the operation of AI that might incorporate other data into decoding neural data, and rigorous cybersecurity will help to make communication neurotechnology suitable for use in higher consequence contexts so important to important personal decisions.

In sum, an understanding of disability as resulting from the interaction of biological and social factors (World Health Organization, 2001; Bickenbach, 2012), invites us to look both at the communication technology and at the environment within which it will be deployed. This dual approach will more realistically do justice to the importance that high consequence communications such as legal testimony and important medical decision-making have for people. Furthermore, the potential benefits of communication neurotechnologies will not be possible unless they are realistically accessible. Human rights obligations such as those set out in the CRPD underscore the importance of social policies supporting development of widely accessible communication neurotechnologies that can be used for everyday use but also for personal decisions of high importance. At the same time, the success of these communications neurotechnologies will likely require the adaptation of the legal system and social expectations about what constitutes reliable and autonomous speech.

## Data Availability Statement

The original contributions presented in the study are included in the article/supplementary material, further inquiries can be directed to the corresponding author/s.

## Author Contributions

JC and KV conceptualized the study. JC, KV, SB, and JB conducted the underlying research. JC wrote the first draft of the manuscript. All authors contributed to the revision, read and approved the submitted version.

## Conflict of Interest

The authors declare that the research was conducted in the absence of any commercial or financial relationships that could be construed as a potential conflict of interest.

## Publisher’s Note

All claims expressed in this article are solely those of the authors and do not necessarily represent those of their affiliated organizations, or those of the publisher, the editors and the reviewers. Any product that may be evaluated in this article, or claim that may be made by its manufacturer, is not guaranteed or endorsed by the publisher.
